# Lipopolysaccharides Impair Insulin Gene Expression in Isolated Islets of Langerhans via Toll-Like Receptor-4 and NF-κB Signalling

**DOI:** 10.1371/journal.pone.0036200

**Published:** 2012-04-27

**Authors:** Julie Amyot, Meriem Semache, Mourad Ferdaoussi, Ghislaine Fontés, Vincent Poitout

**Affiliations:** 1 Montreal Diabetes Research Center, Centre de Recherche du Centre Hospitalier de l'Université de Montréal (CRCHUM), Montréal, Quebec, Canada; 2 Department of Biochemistry, Université de Montréal, Montréal, Quebec, Canada; 3 Department of Medicine, Université de Montréal, Montréal, Quebec, Canada; Boston University, United States of America

## Abstract

**Background:**

Type 2 diabetes is characterized by pancreatic β-cell dysfunction and is associated with low-grade inflammation. Recent observations suggest that the signalling cascade activated by lipopolysaccharides (LPS) binding to Toll-Like Receptor 4 (TLR4) exerts deleterious effects on pancreatic β-cell function; however, the molecular mechanisms of these effects are incompletely understood. In this study, we tested the hypothesis that LPS alters insulin gene expression via TLR4 and nuclear factor kappa-light-chain-enhancer of activated B cells (NF-κB) in islets.

**Methodology/Principal Findings:**

A 24-h exposure of isolated human, rat and mouse islets of Langerhans to LPS dose-dependently reduced insulin gene expression. This was associated in mouse and rat islets with decreased mRNA expression of pancreas-duodenum homebox-1 (PDX-1) and mammalian homologue of avian MafA/l-Maf (MafA). Accordingly, LPS exposure also decreased glucose-induced insulin secretion. LPS repression of insulin, PDX-1 and MafA expression, as well as its inhibition of insulin secretion, were not observed in islets from TLR4-deficient mice. LPS inhibition of β-cell gene expression in rat islets was prevented by inhibition of the NF-κB pathway, but not the p38 mitogen-activated protein kinase (p38 MAPK) pathway.

**Conclusions/Significance:**

Our findings demonstrate that LPS inhibit β-cell gene expression in a TLR4-dependent manner and via NF-κB signaling in pancreatic islets, suggesting a novel mechanism by which the gut microbiota might affect pancreatic β-cell function.

## Introduction

The prevalence of diabetes mellitus is rising across the world, closely associated with a dramatic increase in obesity rates. Type 2 diabetes (T2D) is characterized by defective insulin secretion from the pancreatic β-cell and diminished insulin sensitivity in peripheral tissues. According to the “metainflammation” hypothesis, T2D is also considered as a state of chronic, systemic and low-grade inflammation [1]. Circulating levels of several inflammatory mediators such as acute-phase protein, cytokines and markers of endothelial activation are elevated in T2D patients (reviewed in [2]). Pancreatic β-cells are capable, under certain pathological circumstances, of producing the proinflammatory cytokine interleukin-1β (IL-1β) which can in turn impair β-cell function and induce apoptosis [3], [4]. The proof-of-concept that inflammation plays a role in the pathogenesis of T2D has been provided by the results of a clinical trial showing that administration of an IL-1-receptor antagonist (IL-1Ra) improves glycemic control in T2D patients [5].

In recent years, the gut microbiota has been proposed as an environmental factor increasing the risk of metabolic disorders such as T2D, leading to the endotoxemia concept [6]. Accordingly, subjects with T2D present an altered microbiota reported to be enriched in gram-negative bacteria [7], [8] which express lipopolysaccharides (LPS). This is associated with increased circulating levels of LPS and low-grade endotoxemia which plays a role, at least in part, in the onset of metabolic diseases. In support of this, modulation of the gut microflora in rodents reduces circulating levels of LPS and protects from diet-induced glucose intolerance, insulin resistance and inflammation [9]–[13]. Importantly, evidence for this concept is emerging in humans [14], [15].

Circulating LPS bind Toll-Like Receptor 4 (TLR4) and its co-receptors CD14 and MD-2. TLR4 homodimerizes and recruits the adaptor proteins Myeloid differentiation primary response gene 88 (MyD88) and Toll-IL-1 receptor (TIR) domain-containing adapter-inducing interferon-β (TRIF) through their TIR domains, and activates nuclear factor kappa-light-chain-enhancer of activated B cells (NF-κB), p38 mitogen-activated protein kinases (p38 MAPK), activator protein 1 (AP-1) and interferon-inducible inflammatory gene expression. TLR4 is present in antigen-presenting cells, but also in non-immune cells such as adipocytes [16], myocytes [17] and pancreatic β-cells [18]–[20]. Interestingly, recent evidence suggests that activation of TLR4 signalling can induce both insulin resistance and pancreatic β-cell dysfunction. Thus, deletion or mutation of the gene encoding TLR4 was shown to protect against fatty acid-induced insulin resistance and diet-induced obesity [16], [17], [21]–[23]. In addition, LPS inhibit insulin secretion and insulin gene expression in isolated islets of Langerhans and in β-cell lines [18]–[20]. However, the precise molecular and signalling mechanisms by which LPS affect β-cell function are unknown.

The aim of this study was to determine the molecular mechanisms by which LPS impair insulin gene expression, and to test the hypothesis that this effect involves TLR4 and NF-κB signalling.

## Results

### LPS impair insulin pre-mRNA expression in isolated rat and human islets

**Figure 1:**
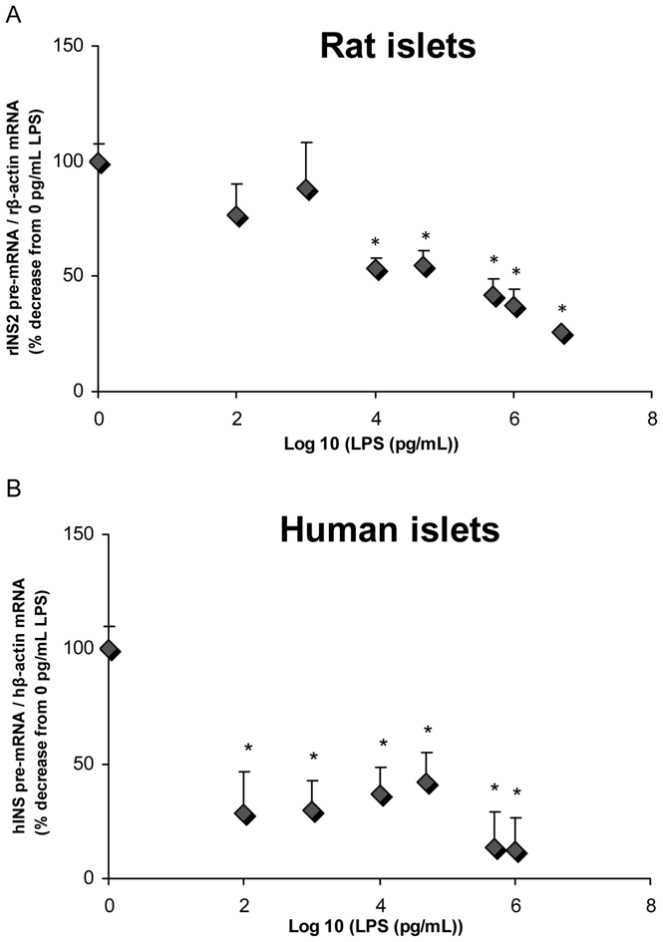
Exposure to LPS dose-dependently represses insulin pre-mRNA expression in isolated rat and human islets. Insulin pre-mRNA levels in response to increasing doses of LPS in isolated rat (A) and human (B) islets. Pre-mRNA levels were measured by RT-PCR and normalized to β-actin mRNA levels. Data are mean ± S.E.M. of 2–6 independent experiments; *p<0.05 vs 0 pg/mL.

First we examined the effects of LPS on insulin gene expression in isolated islets. To this aim we measured insulin pre-mRNA, which has a much shorter half-life than mature insulin mRNA and more directly reflects insulin transcription rates [24]. Isolated rat (Fig. 1A) and human (Fig. 1B) islets were exposed for 24 h to increasing concentrations of LPS. LPS dose-dependently decreased insulin 2 pre-mRNA expression in rat isolated islets (Fig. 1A; n = 3–6; p<0.05). Human islets were much more sensitive to LPS than rat islets, with a significant decrease in human insulin pre-mRNA expression observed at 0.1 ng/mL (Fig. 1B; n = 2–6; p<0.05). At the highest concentration of LPS used in these experiments (5 µg/ml), we could not detect any cell death by caspase-3/7 activity (data not shown). These results indicate that LPS inhibit insulin gene expression in isolated islets.

### Exposure to LPS represses insulin, pancreas-duodenum homeobox-1 (PDX-1) and mammalian homolog of avian MafA/L-Maf (MafA) gene expression via TLR4

**Figure 2:**
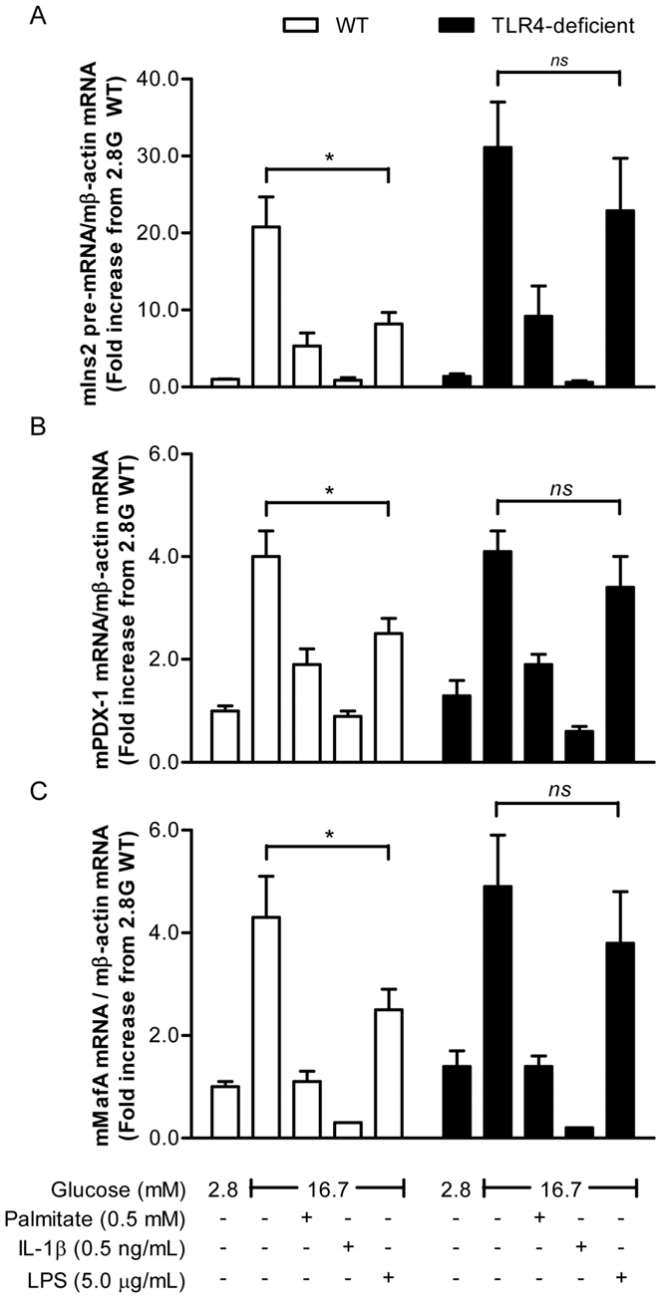
Exposure to LPS decreases insulin, PDX-1 and MafA gene expression in isolated islets via TLR4. (A) Insulin pre-mRNA expression (B) PDX-1 mRNA expression and (C) MafA mRNA expression in islets isolated from WT and TLR4-deficient mice exposed for 24 h to 2.8 (2.8 G) and 16.7 mM (16.7 G) glucose in the presence or absence of 0.5 mM palmitate (PA), 0.5 ng/mL IL-1β or 5 µg/mL LPS. mRNA levels were measured by RT-PCR and normalized to β-actin mRNA levels. Data are mean ± S.E.M. of 6 independent experiments; *p<0.05.

To investigate the role of TLR4 in the inhibitory effects of LPS on the insulin gene, islets from TLR4-deficient C3H/HeJ mice, which harbour a missense mutation in the *tlr4* gene rendering TLR4 unable to signal in response to LPS, and wild-type (WT) C3H/HeOuJ littermates were isolated and exposed for 24 h to 2.8 or 16.7 mM glucose in the presence or absence of 5 µg/mL LPS. Palmitate (0.5 mM) and IL-1β (0.5 ng/mL) were used as positive controls. As expected, exposure to glucose increased insulin 2 pre-mRNA levels, and this was greatly reduced in the concomitant presence of palmitate or IL-1β, both in WT and TLR4-deficient islets. Consistent with the results shown in Fig. 1A, LPS reduced insulin 2 pre-mRNA expression in WT islets (Fig. 2A; n = 6; p<0.05). However, no significant reduction in insulin 2 pre-mRNA was observed in response to LPS in TLR4-deficient islets (Fig. 2A).

Insulin gene expression in adult β-cells is regulated, amongst others, by the glucose-responsive transcription factors PDX-1 and MafA [25]. To examine whether LPS inhibition of insulin gene expression involves these transcription factors, we measured their mRNA levels in islets from WT and TLR4-deficient mice cultured under similar conditions as above. As expected, both palmitate and IL-1β suppressed PDX-1 and MafA mRNA expression in both WT and TLR4-deficient mice (Fig. 2B & C). LPS inhibited both PDX-1 and MafA mRNA levels in islets isolated from WT mice, but not in islets isolated from TLR4-deficient mice. Taken together, these data demonstrate that LPS repress insulin, PDX-1 and MafA gene expression in islets and that this effect requires TLR4.

### Exposure to LPS does not affect PDX-1 nuclear localization

**Figure 3:**
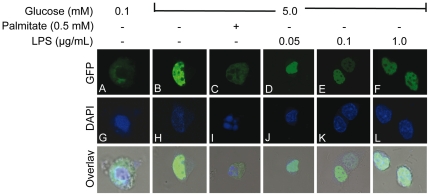
PDX-1 cellular localization is not altered in response to LPS. HIT-T15 cells were transfected with a construct encoding a PDX-1-GFP fusion protein. PDX-1 localization (green) (A–F) was visualized by GFP fluorescence using a laser-scanning confocal microscope in cells cultured in 0.1 and 5 mM glucose with or without 0.5 mM palmitate or increasing doses of LPS. 4′,6-diamidino-2-phenylindole (DAPI) (blue) was used for nuclear staining (G–L). Images are representative of 3 replicate experiments.

Chronic exposure to palmitate inhibits insulin gene expression in islets in part via exclusion of PDX-1 from the nuclear compartment [26]–[28]. To examine whether LPS act via a similar mechanism, a PDX-1-GFP construct was overexpressed in HIT-T15 insulin-secreting cells and exposed to 0.1 mM glucose or 5.0 mM glucose in the presence or absence of 0.5 mM palmitate or increasing doses of LPS for 24 h. PDX-1 expression and subcellular localization were visualized using GFP fluorescence under a confocal microscope (Fig. 3A–F). Nuclei were visualized using DAPI staining (Fig. 3G–L). As expected, PDX-1 expression at 0.1 mM glucose was low in intensity and restricted to the cytosol (Fig. 3A&G). In response to high (5.0 mM) glucose, PDX-1 was predominantly localized in the nucleus (Fig. 3B&H). As we previously observed [27], [28], addition of palmitate restricted PDX-1 localization to the cytosolic compartment (Fig. 3C&I). In contrast, exposure of HIT-T15 cells to increasing doses of LPS did not affect PDX-1 nuclear localization (Fig. 3D–F&J–L). This suggests that LPS does not alter PDX-1 subcellular localization.

### LPS inhibit insulin secretion in WT but not TLR4-deficient mice

**Figure 4:**
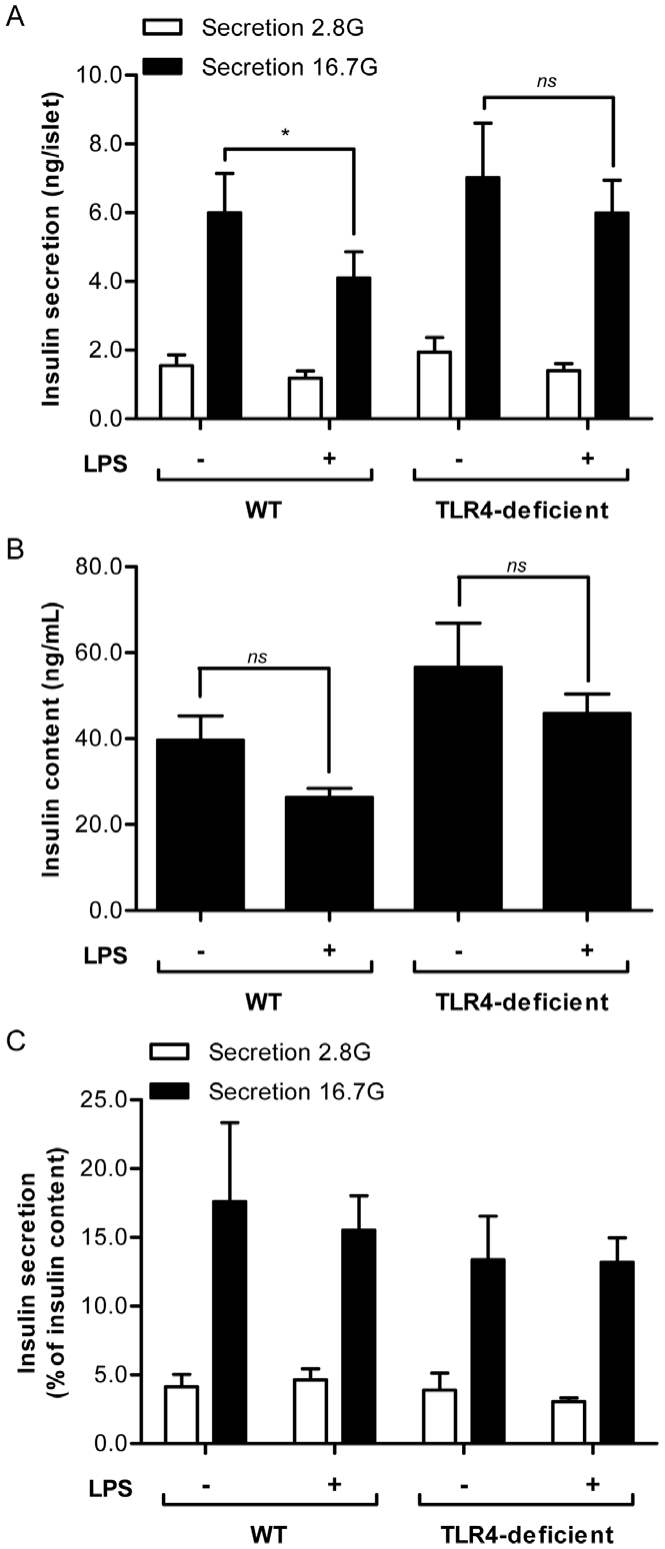
Insulin secretion is reduced in response to LPS in WT but not TLR4-deficient mouse islets. Insulin secretion (A), insulin content (B) and insulin secretion normalized by insulin content (C) as assessed in 1-h static incubations of isolated WT and TLR4-deficient islets at basal (2.8 mM) and stimulatory (16.7 mM) glucose following a 24-h exposure to 16.7 mM glucose in the presence or absence of 5 µg/mL LPS. Data are mean ± S.E.M. of 5 independent experiments; *p<0.05.

LPS have been reported to inhibit glucose-induced insulin secretion (GSIS) in isolated islets [18], [20]. To assess the role of TLR4 in the impairment of GSIS by LPS, islets from WT and TLR4-deficient mice were cultured for 24 h at 16.7 mM glucose in the presence or absence of 5 µg/mL LPS, after which GSIS was measured in 1-h static incubations (Fig. 4). Basal insulin secretion was not significantly different between WT and TLR4-deficient islets in the absence or presence of LPS. Stimulated insulin release (in response to 16.7 mM glucose) from WT islets was slightly but significantly lower in the presence of LPS (Fig. 4A; n = 5; p<0.05). Moreover, this effect appeared to require the presence of TLR4 since GSIS from islets isolated from TLR4-deficient mice was not affected by LPS (Fig. 4A; n = 5; ns). Insulin content from islets isolated from both genotypes trended lower after LPS exposure (Fig. 4B), such that when normalized to intracellular insulin content, the difference in GSIS between LPS-exposed and control WT islets was no longer significant (Fig. 4C). These results suggest that LPS decrease GSIS and that this effect might be in part due to reduction in intracellular insulin stores.

### LPS inhibition of insulin, PDX-1, and MafA gene expression is prevented by inhibition of NF-κB but not p38 MAPK

**Figure 5:**
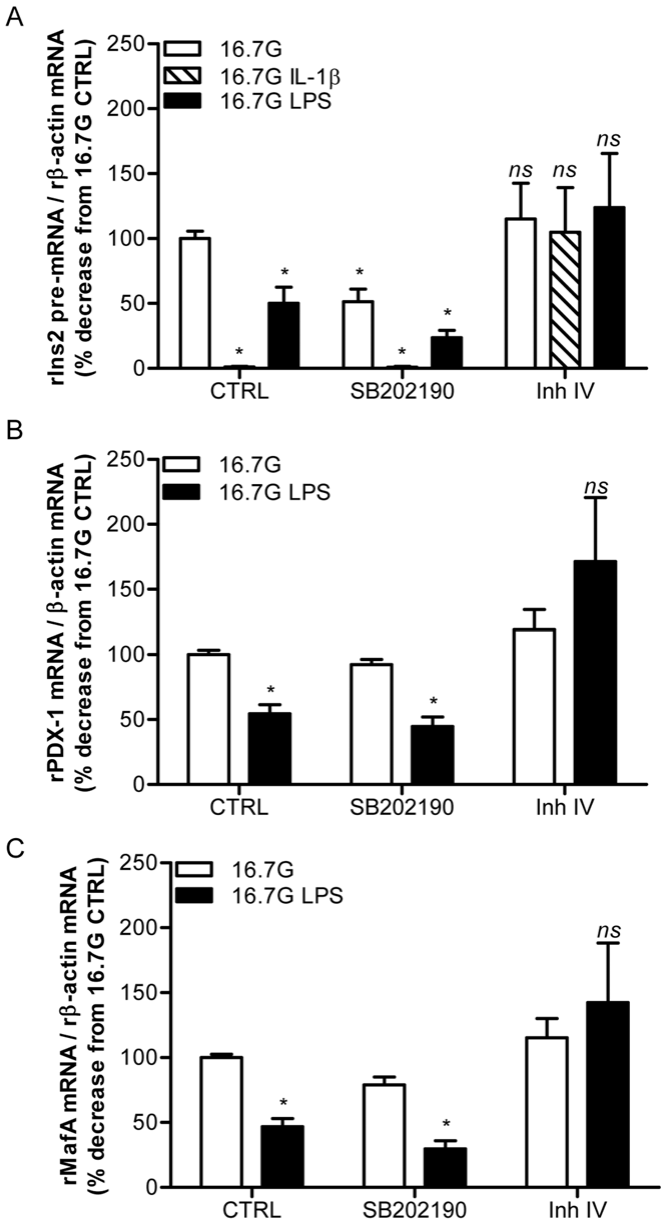
Inhibition of NF-κB, but not p38 MAPK, restores insulin, PDX-1, and MafA gene expression in islets exposed to LPS. (A) Insulin pre-mRNA, (B) PDX-1 mRNA and (C) MafA mRNA expression in isolated rat islets exposed for 24 h to 16.7 mM (16.7 G) glucose in the presence or absence of 10 ng/mL LPS or 0.5 ng/mL IL-1β with or without SB202190 (10 µM) and IKK-2 Inh IV (10 µM). Data are mean ± S.E.M. of 3–4 independent experiments; *p<0.05.

TLR4 signalling involves both the NF-κB and p38 MAPK pathways. To examine the implication of these pathways in LPS inhibition of β-cell gene expression, we used the soluble p38 MAPK inhibitor SB202190 (10 µM) and the NF-κB inhibitor IKK-2 Inhibitor IV (10 µM) (Fig. 4) in isolated rat islets cultured for 24 h at 16.7 mM glucose in the presence or absence of 10 ng/mL LPS. IL-1β (0.5 ng/mL) was used as a positive control known to signal via NF-κB [3]. As observed in Fig. 2 in mouse islets, both LPS and IL-1β decreased insulin 2 pre-mRNA expression in rat islets (Fig. 5A). This was completely prevented in the presence of the NF-κB inhibitor, but not of the p38 MAPK inhibitor (Fig. 5A; n = 3–4; p<0.05). In addition, the NF-κB inhibitor, but not the p38 MAPK inhibitor, completely prevented the decrease in PDX-1 (Fig. 5B; n = 4; p<0.05) and MafA (Fig. 5C; n = 4; p<0.05) expression in response to LPS. Using nuclear extracts, we also examined NF-κB nuclear expression by immunoblotting in rat and human isolated islets exposed for 24 h to 16.7 mM glucose in the presence or the absence of 50 or 100 ng/mL LPS. Consistent with the results shown in Fig. 5, nuclear extracts from both rat and human islets exposed to LPS were enriched in the p65 subunit of NF-κB (Fig. S1A&B; n = 3; p<0.05). In addition, expression of the NF-κB target gene TNF-α was increased in rat islets after a 24-h exposure to 10 ng/mL LPS (Supp.Fig. 1C; n = 4; p<0.05), confirming activation of the pathway. These data therefore demonstrate that LPS inhibition of insulin, PDX-1, and MafA gene expression in islets requires TLR4 and NF-κB signalling.

## Discussion

TLRs are one of the most ancient and conserved components of the innate immune system and play key roles in recognizing pathogen-associated molecules such as LPS. As many as 12 and 10 different isoforms of TLRs have been identified in humans and rodents, respectively. Pancreatic β-cells express significant levels of TLR4 which render them sensitive to LPS [18]–[20]. This study was designed to identify the molecular mechanisms underlying the deleterious effects of LPS on pancreatic β-cell function. Our results uniquely demonstrate the requirement for TLR4 and NF-κB signalling in LPS impairment of β-cell gene expression.

Insulin gene expression, essentially restricted to pancreatic β-cells in adults, is tightly regulated by a highly sophisticated transcriptional network. In response to stimulatory glucose conditions, PDX-1, MafA and BETA2/NeuroD bind respectively to the A3, C1 and E1 cis-acting DNA elements on the proximal region of the insulin promoter and activate transcription in a synergistic and coordinated manner (reviewed in [25], [29]). In addition, glucose stimulates MafA expression [30] and promotes PDX-1 translocation from the cytoplasm to the nucleus [31]. On the other hand, insulin gene expression is altered under pathological circumstances, such as chronically excessive levels of glucose or fatty acids [32]. Thus, elevated fatty acids inhibit insulin gene expression via impaired PDX-1 nuclear localization and decreased binding of PDX-1 and MafA to the insulin promoter [27], [28], [33].

In the present study, we confirmed that LPS impair insulin gene expression in human islets [18]. Importantly, we found human islets to be much more sensitive to LPS than rat islets, as insulin pre-mRNA levels were significantly decreased in human islets at the lowest dose of LPS used in this study (0.1 ng/mL). Since circulating LPS concentrations are reported to be in the ng/mL range in endotoxemia associated with metabolic diseases [15], our results suggest that LPS inhibition of insulin gene expression might occur in vivo in humans.

Using isolated islets from a mouse line harboring a TLR4 mutation that renders it unresponsive to LPS, we further showed, to our knowledge for the first time, that TLR4 is required for the effects of LPS, but not those of palmitate, on insulin gene expression. This is in contrast to a recent study reporting a role for TLR4 in mediating palmitate-induced apoptosis in insulin-secreting INS1 cells [34], a discrepancy which might be explained by different mechanisms underlying fatty-acid cytotoxicity vs. their effects on the insulin gene, and/or differences between cell lines and primary islets. We have previously shown that exposure of isolated islets to exogenous palmitate leads to an increase in intracellular ceramide content via *de novo* synthesis [33]. Ceramide generation has recently been shown to mediate saturated fatty acid-induced insulin resistance in skeletal muscle via TLR4 signalling [35]. In contrast, our results showing that palmitate inhibition of insulin gene expression still occurs in the absence of a functional TLR4 indicate that TLR4 signalling is dispensable for fatty-acid-induced β-cell dysfunction. Our results also show that LPS reduce PDX-1 and MafA mRNA expression in a TLR4-related manner without affecting PDX-1 nuclear localization. This mechanism of action is therefore different from that of palmitate, which affects PDX-1 and MafA expression as well as PDX-1 nuclear translocation ([27], [28] and Fig. 3).

It has been previously shown that exposure of isolated islets or β-cell lines to LPS decreases insulin secretion [18], [20]. We confirmed that GSIS was reduced in isolated mouse islets in response to LPS and showed that this requires a functional TLR4. Our results are consistent with previous studies [18], [20], but not with a recent report by Kiely et al. [19] in insulin-secreting BRIN-BD11 cells. This discrepancy might be explained by differences between clonal cells and primary islets. Alternatively, the net effect of LPS on insulin secretion might depend on whether or not the results are normalized to insulin content. Indeed, in our study GSIS was no longer significantly altered by LPS when expressed as a percentage of intracellular insulin content, suggesting that inhibition of insulin biosynthesis might account, at least in part, for the reduced secretion, although this remains to be directly tested. Finally, it is unlikely that the observed impairment of insulin gene expression and insulin secretion in response to LPS merely results from β-cell death, since we did not observed any activation of caspase-3/7 under our experimental conditions.

**Figure 6:**
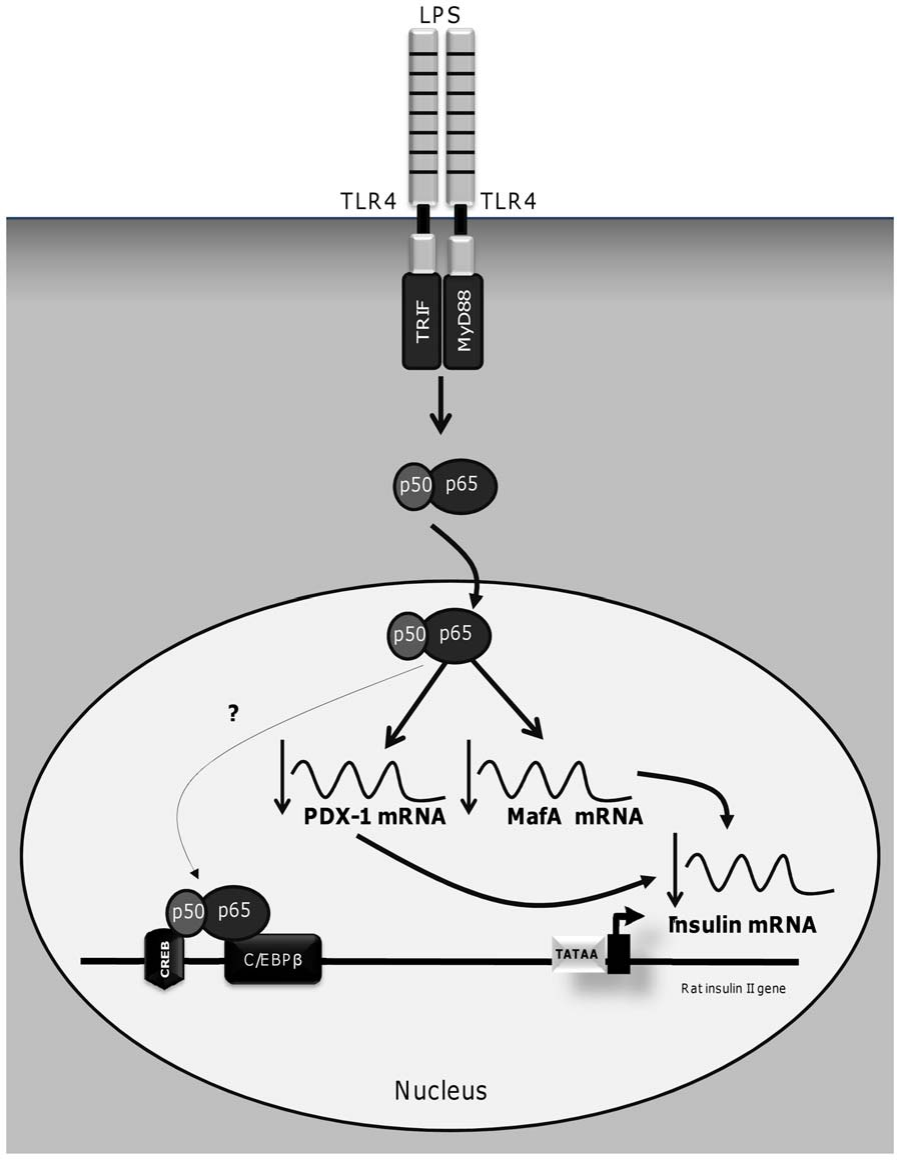
Potential mechanism by which LPS repress insulin gene expression in isolated islets. Exposure to LPS activates the NF-κB pathway in isolated islets and inhibits the expression of insulin, PDX-1 and MafA. The decrease in insulin expression might indirectly results from LPS inhibition of PDX-1 and MafA. NF-κB could also inhibit insulin gene expression by interacting with other proteins such as C/EBPβ and/or CREB, as observed in other cell types.

The transcription factor NF-κB plays a central role in the innate immune response and inflammation [36]. NF-κB is normally retained in the cytoplasm via its binding to the inhibitor protein κB (IκB). In response to stimuli such as IL-1β and LPS, the interaction between inhibitor kinase (IKK) complex and IκB leads to its phosphorylation. IκB is subsequently ubiquitinated and degraded, enabling the release and translocation of NF-κB to the nucleus and subsequent activation of its target genes such as TNF-α [37], [38]. In this study, we demonstrate that LPS exposure increases NF-κBp65 nuclear localization in both rat and human islets. We further showed that pharmacological suppression of the NF-κB pathway completely restores insulin, PDX-1 and MafA gene expression upon LPS exposure. The question then arises as to how does NF-κB repress insulin gene expression in response to LPS? In pancreatic β-cells, NF-κB is a key regulator of β-cell survival and function [39]–[42]. Cytokine-induced NF-κB activation has been reported to decrease PDX-1 mRNA expression [43]. Here, we report that LPS-induced activation of NF-κB reduces insulin, PDX-1 and MafA mRNA levels. Thus, the reduction in insulin gene expression by NF-kB could be indirectly due to its inhibition of the two key transcription factors PDX-1 and MafA (Fig. 6). Since there are no known NF-κB binding sites on the insulin, PDX-1 or MafA promoters, inhibition of these genes by NF-κB might be mediated through interactions with other proteins. For instance, we observed that LPS increases CCAAT-enhancer-binding protein β (C/EBPβ) mRNA expression in isolated islets from WT mice, but not in TLR4-deficient mice (data not shown). It is therefore conceivable that the p65 subunit of NF-κB might interact with C/EBPβ, a known repressor of the insulin gene [44]–[46], and increase C/EBPβ binding to the insulin promoter, as seen in other cell types [47], [48], thereby repressing insulin gene transcription. Another factor known to interact with NF-κBp50 is the cAMP response element (CRE)-binding protein (CREB) [49]. Since CREB induces insulin gene transcription (reviewed in [50]), it is conceivable that an interaction between CREB and NF-κB might prevent the binding of CREB to the insulin promoter.

Our results reveal a role for TLR4 in LPS-mediated β-cell dysfunction. TLR4 signalling has recently been reported to impair insulin secretion and insulin mRNA expression in human islets via its activation by the chemokine CXCL10 [51], but the effects of LPS were not examined. On the other hand TRIF, a critical molecule in the TLR signalling pathway, was recently shown to be important for normal β-cell function [52]. Thus, TRIF-null mice exhibit hyperglycemia, impaired glucose tolerance, and defective GSIS [52]. This suggests that TRIF might be required for normal β-cell function but might become deleterious when chronically activated in response to LPS. A similar dual effect has been reported for NF-κB [53].

Finally, pancreatic β-cells express significant levels of TLR2 [20] which can recognize bacterial lipopeptides [54], including LPS [55], [56]. Moreover, TLR2 expression is induced by LPS [57] and it has been recently proposed that up-regulation of TLR2 by low levels of bacterial products can contribute to the mechanisms by which the immune system increases its response to an infection [58]. It is therefore possible that TLR2 amplifies TLR4 signalling in response to LPS and might have contributed some of the effects observed in this study, although this remains to be directly examined.

In conclusion, this study uniquely demonstrates that impairment of insulin gene expression by LPS involves decreased PDX-1 and MafA mRNA levels and requires TLR4 and NF-κB signalling. Importantly, the effects of LPS on the insulin gene in human islets were observed at concentrations similar to the circulating levels achieved during endotoxemia, suggesting that direct repression of the insulin gene might contribute to the metabolic disturbances associated with alterations of the microbiota.

## Materials and Methods

### Reagents

RPMI-1640, fetal bovine serum (FBS) and 4′,6-diamidino-2-phenylindole (DAPI) were obtained from Invitrogen (Burlington, ON, Canada). PBS was obtained from Multicell. Fatty acid–free bovine serum albumin (BSA) was from Equitech-Bio (Kerrville, TX, USA). SB202190 and IKK-2 Inhibitor IV were from Calbiochem (EMD Biosciences, San Diego, CA, USA). Palmitate (sodium salt), LPS (from *Escherichia coli* O111:B4) and all other reagents (analytical grade) were from Sigma unless otherwise noted.

### Islet isolation and culture

Human islets - The use of human islets was approved by the Institutional Ethics Committee of the Centre Hospitalier de l'Université de Montréal (protocol #: ND-05-035). Isolated islets from non-diabetic human cadaveric donors were obtained from the NIH/NIDDK-supported Integrated Islet Distribution Program (http://www.iidp.coh.org), and from the Clinical Islet Laboratory at the University of Alberta. A written consent for research from donor family was obtained for all donors.

Rodent islets - All procedures were approved by the Institutional Committee for the Protection of Animals at the Centre Hospitalier de l'Université de Montréal (protocol #: An09027VPr). 8 week-old WT (C3H/HeOuJ) and TLR4-deficient (C3H/HeJ) mice (harboring a missense mutation in the *tlr4* gene rendering TLR4 unable to signal in response to LPS) were obtained from the Jackson Laboratories (Bar Harbor, Maine, USA). 250–275 g male Wistar rats were obtained from Charles River (St.-Constant, QC, Canada). Animals were housed under controlled temperature (21°C) and a 12-h light-dark cycle with free access to water and standard laboratory chow. Mice and rats were anesthetized by IP injection of a Ketamine Hydrochloride (Bimeda-MTC Animal Health Inc., Cambridge, ON)/Xylazine (Bayer Inc., Toronto, ON) mixture and islets were isolated by collagenase digestion and dextran density gradient centrifugation as described [59].

Culture - Isolated islets were cultured in RPMI 1640 containing 10% FBS and exposed for 24 h to 2.8 or 16.7 mM glucose in the presence or the absence of palmitate, IL-1β or LPS. Preparation of the culture media containing palmitate was described previously [33]. The final molar ratio of palmitate:BSA was 5∶1. HIT-T15 cells (passage 75–78; kindly provided by R.P. Robertson (Pacific Northwest Diabetes Research Institute, Seattle, WA, USA)) were maintained in RPMI-1640 media containing 10% FBS and 11.1 mM glucose.

### RNA extraction and real-time RT-PCR

Total RNA was extracted from aliquots of 150 islets each using the RNeasy Qiagen micro-kit (Qiagen Inc., Mississauga, ON), reverse transcribed, and real-time RT-PCR was carried out using the Quantitect SYBR Green PCR Kit (Qiagen Inc., Mississauga, ON), as previously described [28]. To amplify preproinsulin pre-mRNA (Ins pre-mRNA), a forward primer was designed against a sequence in exon 2 and a reverse primer designed against a sequence in intron 2, as described [24]. Primers used for real-time RT-PCR are listed in Table S1. All primer sets were designed using Primer3 [60]. Results are expressed as the ratio of target mRNA to β-actin mRNA.

### PDX-1-GFP plasmids, transient transfections and immunohistochemistry

A vector encoding a PDX-1-GFP fusion protein was generated from a PDX1-cMyc construct [31]. Briefly, after stop codon removal from the initial PDX1-cMyc sequence, the amplified 906-nucleotide fragment was restricted by HindIII and KpnI, and ligated into the mammalian expression vector pcDNA3.1-GFP in line with the open–reading frame. For transient transfections, HIT-T15 cells (passages 75 to 78) were seeded in 24-well plates at a density of 80 000 cells/well 2 days before transfection. Cells were transfected with a total of 0.8 µg PDX-1-GFP DNA and 2 µL Lipofectamine 2000. Cells were fixed 24 h following treatments (see results) with 3.7% formaldehyde for 30 min and permeabilized with 0.1% Triton X-100 for 10 min and cold methanol for 20 min. After blocking with 5% normal horse serum, cells were incubated with 10 µg/mL DAPI. Cells were observed under a Leica TCS SP5 confocal microscope (63× Oil) (Leica Microsystems [Canada], Richmond Hill, ON).

### Nuclear extracts and Western blot analyses

Nuclear proteins (10 µg) from isolated rat or human islets were prepared and subjected to 10% SDS PAGE as previously described [27]. Immunoblots were performed with anti-NF-κBp65 (Cell signalling, Boston, MA, USA) and anti-histone deacetylase 1 (HDAC1) (Millipore, Billerica, MA, USA) antibodies. Signals were detected using a horseradish peroxidase-labeled anti-rabbit IgG (BioRad) and enhanced chemiluminescence (ECL, PerkinElmer Las Canada Inc.,Woodbridge, ON) on Kodak films (Kodak, Rochester, NY, USA).

### Caspase-3/7 activity assay

Caspase-3/7 activity was measured using a kit (Promega, Madison, WI, USA) according to the manufacturer's instructions. Briefly, 25 islets were cultured for 24 h with increasing doses of LPS (1 ng/mL to 5 µg/mL), and then incubated for 30 min. with the Caspase-Glo 3/7 reagent. Fluorescence was measured using a FluoStar-Optima microplate reader as described [61]. Results were normalized per islet.

### Insulin secretion in isolated islets

Insulin secretion was assessed in 1-h static incubations. Batches of 10 islets each were washed twice in Krebs-Ringer buffer containing 0.1% BSA and 2.8 mM glucose for 20 min at 37°C, then incubated for 1 h at 37°C in either 2.8 or 16.7 mM glucose. Each condition was run in triplicate. Intracellular insulin content was determined after acidified-ethanol extraction. Insulin was measured by radioimmunoassay (LINCO Research, St. Charles, MO, USA).

### Statistical analysis

Data are expressed as mean ± SEM and were analyzed by unpaired t-test or ANOVA with Dunnett's or Bonferroni post-hoc adjustments for multiple comparisons, as appropriate. p<0.05 was considered significant.

## Supporting Information

Figure S1 **LPS activate NF-κB signaling and TNF-α mRNA expression in isolated rat islets.** (A) Representative immunoblot of nuclear extracts using antibodies against NF-κBp65 and HDAC1 in rat islets exposed for 24 h to 16.7 mM (16.7 G) glucose in the presence or absence of 100 ng/mL LPS, or human islets exposed for 24 h to 16.7 mM (16.7 G) glucose in the presence or absence of 50 ng/mL LPS. (B) Quantification of NF-κBp65 nuclear expression in rat islets exposed for 24 h to 16.7 mM (16.7 G) glucose in the presence or the absence of 100 ng/mL LPS (n = 3) (C) TNF-α mRNA expression in rat isolated islets exposed for 24 h to 16.7 mM (16.7 G) glucose in the presence or the absence of 10 ng/mL LPS or 0.5 ng/mL IL-1β. Data are mean ± S.E.M. of 4 independent experiments. *p<0.05.
(TIF)

Table S1 **Primer sequences for real-time RT-PCR.**
(DOC)
