# Supplementary figures and images for: Lipopolysaccharides Impair Insulin Gene Expression in Isolated Islets of Langerhans via Toll-Like Receptor-4 and NF-κB Signalling
Source: PLoS One. 2012 Apr 27;7(4):e36200. doi: 10.1371/journal.pone.0036200 (PMC3338606; doi:10.1371/journal.pone.0036200)

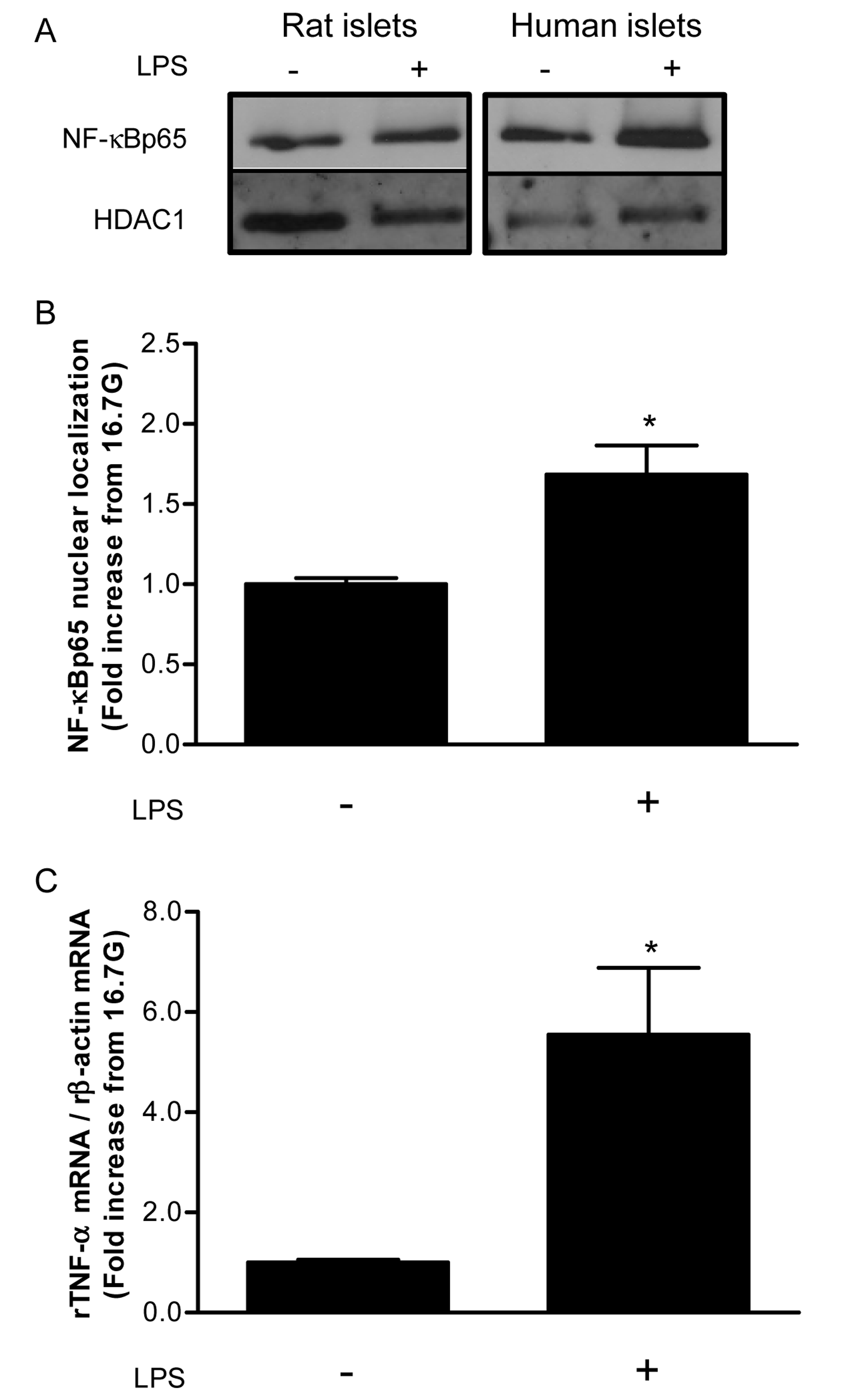

Supplement: Figure S1 — LPS activate NF-κB signaling and TNF-α mRNA expression in isolated rat islets. (A) Representative immunoblot of nuclear extracts using antibodies against NF-κBp65 and HDAC1 in rat islets exposed for 24 h to 16.7 mM (16.7 G) glucose in the presence or absence of 100 ng/mL LPS, or human islets exposed for 24 h to 16.7 mM (16.7 G) glucose in the presence or absence of 50 ng/mL LPS. (B) Quantification of NF-κBp65 nuclear expression in rat islets exposed for 24 h to 16.7 mM (16.7 G) glucose in the presence or the absence of 100 ng/mL LPS (n = 3) (C) TNF-α mRNA expression in rat isolated islets exposed for 24 h to 16.7 mM (16.7 G) glucose in the presence or the absence of 10 ng/mL LPS or 0.5 ng/mL IL-1β. Data are mean ± S.E.M. of 4 independent experiments. *p<0.05. (TIF) [file pone.0036200.s001.tif]
