# Supplementary material for: Lipopolysaccharides Impair Insulin Gene Expression in Isolated Islets of Langerhans via Toll-Like Receptor-4 and NF-κB Signalling
Source: PLoS One. 2012 Apr 27;7(4):e36200. doi: 10.1371/journal.pone.0036200 (PMC3338606; doi:10.1371/journal.pone.0036200)
Supplement: Table S1 — Primer sequences for real-time RT-PCR. (DOC) [file pone.0036200.s002.doc]

| **Target** | **Sense primer (5’-3’)** | **Anti-sense primer (5’-3’)** |
| --- | --- | --- |
| **Human** | | |
| hIns pre-mRNA | tgactgtgtcctcctgtgtcc | acaatgccacgttctgc |
| hβ-actin | ggacttcgagcaagagatgg | agcactgtgttggcgtacag |
| **Rat** |  |  |
| rIns2 pre-mRNA | agcgtggattcttctacacacc | aaggattctcactccttcttgg |
| rβ-actin | acgaggcccagagcaaga | ttggttacaatgccgtgttca |
| rPDX-1 | gaaccggaggagaataagagg | agtcaagttgagcatcactgc |
| rMafA | aggaggaggtcatccgactg | cttctcgctctccagaatgtg |
| **Mouse** |  |  |
| mIns2 pre-mRNA | tgtgtccatccatgaccagt | cagtgccaaggtctgaaggt |
| mPDX-1 | ggtatagccggagagatgc | ctggtccgtattggaacg |
| mMafA | atcaccatcaccaccatcac | tgacctcctccttgctgaag |
| mβ-actin | catggatgacgatatcgctgc | gtacgaccagaggcatacagg |
